# Lower number of modifiable risk factors was associated with reduced atrial fibrillation incidence in an 18-year prospective cohort study

**DOI:** 10.1038/s41598-022-13434-4

**Published:** 2022-06-02

**Authors:** Mi Kyoung Son, Dae Sub Song, Kyoungho Lee, Hyun-Young Park

**Affiliations:** 1 Division of Population Health Research, Department of Precision Medicine, Korea National Institute of Health, 200 OsongSaengmyeong 2-Ro, Osong-Eup, Cheongju, Chungcheongbuk-do 28160 Republic of Korea; 2 Department of Precision Medicine, Korea National Institute of Health, 187 OsongSaengmyeong 2-Ro, Osong-Eup, Cheongju, Chungcheongbuk-do 28159 Republic of Korea

**Keywords:** Epidemiology, Cardiovascular diseases

## Abstract

Prevention strategies for atrial fibrillation (AF) are lacking. This study aimed to identify modifiable risk factors (MRFs) and estimate their impact on AF in the midlife general population. We assessed 9049 participants who were free of prevalent AF at baseline from the Korean Genome and Epidemiology Study. Cox models with time-varying assessment of risk factors were used to identify significant MRFs for incident AF. The MRF burden was defined as the proportion of visits with MRFs during follow-up. Over a median follow-up of 13.1 years, 182 (2.01%) participants developed AF. Three MRFs, including systolic blood pressure (SBP) ≥ 140 mmHg, obesity with central obesity, and an inactive lifestyle were significantly associated with incident AF. Among participants with 3, 2, 1, and 0 MRFs at baseline, 16 (3.9%), 51 (2.5%), 90 (1.8%) and 25 (1.5%) had incident AF, respectively. Compared to participants with three MRFs, those with one or no MRFs had a decreased risk of AF (hazard ratio [95% CI] for one MRF, 0.483 [0.256–0.914]; and for no MRF, 0.291 [0.145–0.583]). A decreasing MRF burden was associated with reduced AF risk (hazard ratio [95% CI] per 10% decrease in burden for SBP ≥ 140 mmHg, 0.937 [0.880–0.997]; for obesity with central obesity, 0.942 [0.907–0.978]; for inactivity, 0.926 [0.882–0.973]). Maintaining or achieving MRF ≤ 1 was associated with decreased AF risk, suggesting that minimizing the burden of MRF might help prevent AF.

## Introduction

Atrial fibrillation (AF), the most common cardiac arrhythmia, is a leading cause of mortality and ischemic stroke^1,2^. The prevalence of AF is expected to increase due to a growing burden of risk factors, such as an aging population, hypertension, obesity, diabetes mellitus and ischemic heart disease^3^. AF also results in significant use of health care resources and causes a substantial economic burden, with AF-related Medicare expenses being approximately $16 billion annually in the United States^4^. Although catheter ablation has been shown to be effective in suppressing AF, it is invasive and has the potential for serious complications despite improved experience and advances in ablation technology^5,6^. Thus, a focus on primary prevention is a critical component of strategies to control the growing burden of AF at a population level.

Many studies of incident AF have focused primarily on risk prediction, AF treatment and AF-related stroke prevention^6–11^. However, there is limited information on preventive strategies to reduce the incidence of AF. In many earlier studies, risk factors for AF were usually assessed at a single time point (at baseline or last before event) and related to outcomes occurring several years, or even decades^8,12–14^. However, the status of a risk factor may change over time due to environmental factors such as physical activity or clinical conditions, and these changes usually become greater with time from exposure assessment. Indeed, true changes in variables over time generally lead to regression dilution bias, resulting underestimation of the true association between exposure and outcome^15^, however regression dilution bias could be addressed by performing time-varying analysis^16^. Previous studies provided the information regarding risk factor changes between these measurements and the appearance of AF^14,17^. However, these results used only two time points (start and end time). For primordial prevention interventions, an appropriate level of control of modifiable risk factors (MRFs) is important.

In the present study, we aimed to identify the MRFs for incident AF using a time-varying model, investigate the cumulative effect of the MRFs burden on AF risk, and provide targets for prevention of AF in a longitudinal, population-based cohort.

## Results

### Baseline characteristics

In 9,049 participants without AF at baseline, 182 participants developed new-onset AF over a median follow-up of 13.1 years (range, 1.4 to 16.6 years). Participants with AF, when compared with people who did not develop AF, were more likely to be older, male, rural, to have cardiovascular disease (CVD), higher waist circumference (WC), higher blood pressure (BP), and lower leisure time physical activity (LTPA) (Table 1).

**Table 1:** Baseline characteristics of study population (n = 9094).

Variables	No AF (N = 8 867)	AF (N = 182)	*P*-value
Age, yrs	52.16 ± 8.9	57.95 ± 8.0	< 0.001
Sex, male	4171 (47.0)	122 (67.0)	< 0.001
Area, rural	4582 (51.7)	118 (64.8)	< 0.001
**Clinical**			
BMI, kg/m^2^	24.58 ± 3.1	25.02 ± 3.4	0.063
WC, cm	82.75 ± 8.8	85.50 ± 8.9	< 0.001
**BMI and WC category**			0.081
Non-obese without central obesity	4577 (51.6)	85 (46.7)	
Obese without central obesity	1632 (18.4)	31 (17.0)	
Non-obese with central obesity	498 (5.6)	7 (3.9)	
Obese with central obesity	2160 (24.4)	59 (32.4)	
Systolic blood pressure, mmHg	121.5 ± 18.2	127.7 ± 20.0	< 0.001
**Systolic blood pressure category, mmHg**			< 0.001
< 120	4496 (50.7)	68 (37.4)	
120–139	2968 (33.5)	72 (39.6)	
≥ 140	1403 (15.8)	42 (23.1)	
Diastolic blood pressure, mmHg	80.30 ± 11.3	82.85 ± 11.46	0.003
LTPA, min/week	75.29 ± 164.5	53.78 ± 122.0	0.021
**LTPA category**			0.206
Inactivity, 0 min/week	6396 (72.1)	139 (76.4)	
Active, > 0 min/week	2471 (27.9)	43 (23.6)	
CKD, eGFR < 60 mL/min	641 (7.2)	16 (8.8)	0.422
CVD	250 (2.8)	15 (8.2)	< 0.001
**Laboratory examinations**			
HbA1c, %	5.79 ± 0.9	5.78 ± 0.8	0.957
Total cholesterol, mg/dL	191.10 ± 35.4	188.00 ± 36.1	0.246

Continuous variables are reported as mean ± standard deviation, and categorical variables are reported as n (%).

AF, atrial fibrillation; BMI, body mass index; WC, waist circumference; CKD, chronic kidney disease; CVD, cardiovascular disease; LTPA, leisure time physical activity.

### Modifiable risk factors for AF

As expected, the incidence rates of AF increased during follow-up (110.10 and 153.55/100,000 person-years in 2003–2004 and 2017–2018, respectively), were consistently higher among males than females, and increased substantially with age (Table S1).

In time-updated multivariable models accounting for changes in risk factors after the baseline survey, three MRFs, systolic blood pressure (SBP) ≥ 140 mmHg, obesity with central obesity, and inactivity, were each significantly associated with the development of incident AF (Table 2). Non-modifiable risk factors associated with new-onset AF included age ≥ 70 years, male sex, and history of CVD. The most significant risk occurred in those with SBP ≥ 140 mmHg (hazard ratio [HR]: 1.539; 95% confidence interval [CI]: 1.007 to 2.350; p = 0.046) when compared with SBP < 120 mmHg and increasing SBP was associated with a significantly increased risk of incident AF (multivariable-adjusted HR: 1.145, 95% CI 1.051 to 1.247 [per 10 mmHg increase]; *p* = 0.002, data not shown). To assess the joint impact of obesity and central obesity, participants were classified into four groups. Obese individuals with central obesity had a higher risk of AF (HR: 1.681; 95% CI 1.194 to 2.366; *p* = 0.003) compared to non-obese individuals without central obesity. Inactivity, when compared with low or high levels of LTPA (> 0 min/weeks), was associated with a 42% higher risk of AF. At baseline, 81.2% of all participants had at least one MRFs associated with AF risk, and the incidence of AF increased markedly with increasing numbers of MRFs (Table S2).

**Table 2:** Risk factors for incident atrial fibrillation using a time-updated model.

	Multivariable-Adjusted HR (95% CI)*	*P*-value
**Modifiable risk factors**		
Systolic blood pressure, mmHg		
< 120	Reference	
120–139	1.215 (0.874–1.688)	0.246
≥ 140	1.539 (1.007–2.350)	0.046
**Combinations of obesity and central obesity**		
Non-obese without central obesity	Reference	
Obese without central obesity	1.166 (0.699–1.945)	0.557
Non-obese with central obesity	1.246 (0.748–2.075)	0.399
Obese with central obesity	1.681 (1.194–2.366)	0.003
**LTPA**		
Active	Reference	
Inactivity	1.420 (1.042–1.936)	0.026
**Other significant risk factors**		
Age, ≥ 70 years	1.653 (1.110–2.462)	0.013
Sex, male	2.635 (1.914–3.628)	< 0.001
Cardiovascular disease	1.841 (1.223–2.771)	0.003

HR, hazard ratio; CI, confidence interval; LTPA, leisure time physical activity.

*Multivariable adjustment was for sex, area and time-updated assessment of age, systolic blood pressure, combinations of obesity and central obesity, LTPA, chronic kidney disease, cardiovascular disease, HbA1c and total cholesterol.

Additionally, assuming a causal relationship between MRFs and AF, 28.7% (95% CI 14.6% to 40.4%) of incident AF in the study population was attributable to these three MRFs, with inactivity being the greatest contributing risk factor (Table 3).

**Table 3:** The population attributable fractions (PAF) and 95% CI of individual risk factors.

Risk factors	PAF (95% CI)
All 3 modifiable risk factors*	28.7 (14.6–40.4)
SBP ≥ 140 mmHg	9.5 (2.4–16.1)
Obesity with central obesity	10.7 (2.2–18.4)
No LTPA (inactivity)	15.6 (2.2–27.2)
Age, ≥ 60 yrs	34.0 (24.3–42.4)
Sex, male	38.4 (27.0–48.1)
CVD	5.8 (1.7–9.8)

CVD, cardiovascular disease; SBP, systolic blood pressure; LTPA, leisure time of physical activity; CI, confidence interval.

*The PAF for all 3 risk factors is numerically smaller than the individual sum of PAF estimates as the summative PAF accounts for overlap in the prevalence of risk factors.

### Optimal levels of modifiable risk factor control for risk reduction of AF

**Figure 1:**
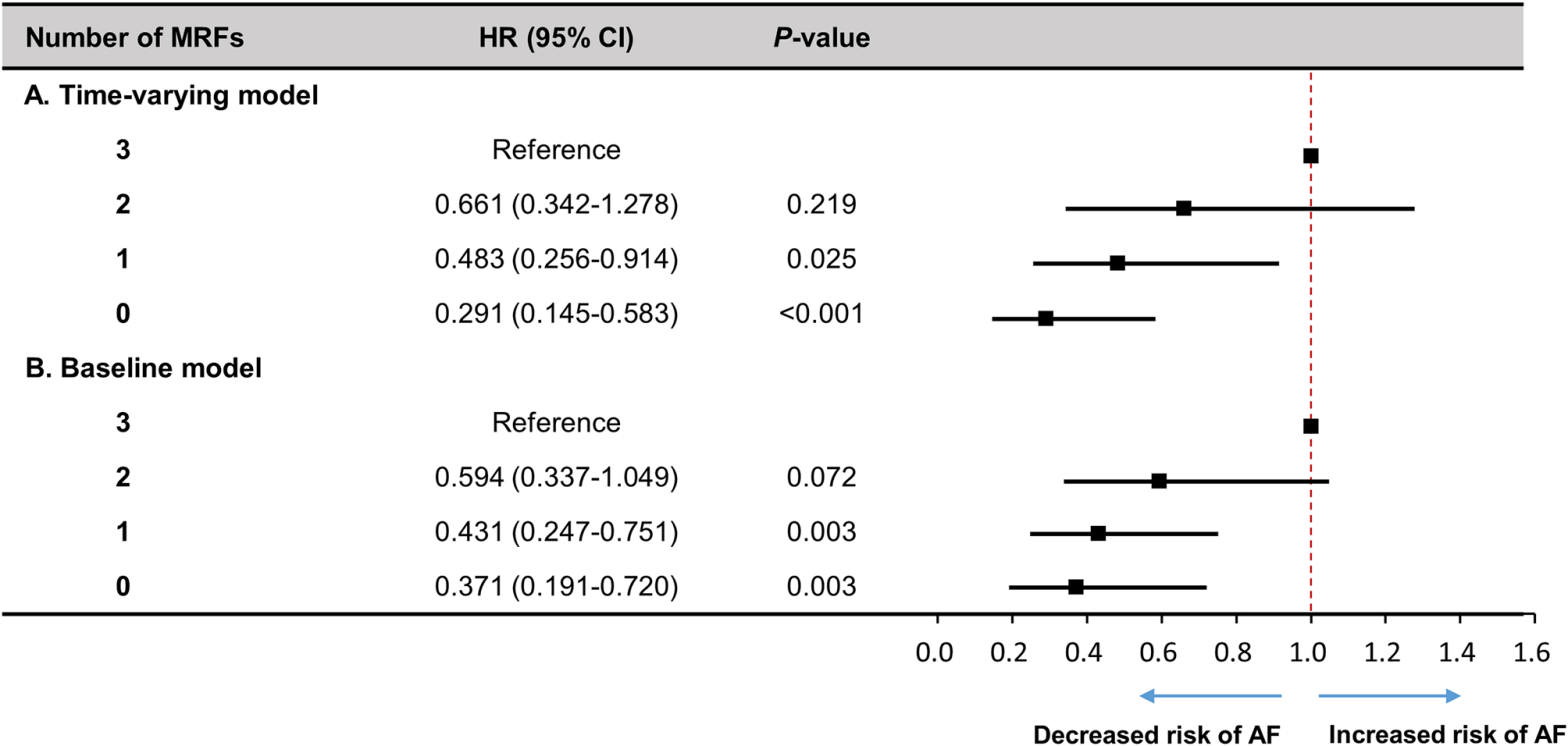
Modifiable risk factors and AF risk in general population using baseline and time-updated models. Modifiable risk factors include time-updated systolic blood pressure ≥ 140 mmHg, obesity with central obesity, and inactivity. Hazard ratios (HR) for the baseline model were adjusted for age, sex, area, chronic kidney disease, cardiovascular disease, HbA1c and total cholesterol at baseline. Time-updated models were adjusted for sex and area at baseline, and time-updated assessment of age, chronic kidney disease, cardiovascular disease, HbA1c and total cholesterol. MRFs = modifiable risk factors; HR = hazard ratio; AF = atrial fibrillation.

To assess the joint impact and find optimal levels of these MRFs to impact on the incidence of AF, we used multivariable adjusted models with time-updated assessment of risk factors after baseline, as well as baseline models (Fig. 1). In the time-updated model, compared with participants with three MRFs, those with MRFs ≤ 1 had a decreased risk of AF (hazard ratio [95% CI] for one MRFs, 0.483 [0.256–0.914]; and for no MRFs, 0.291 [0.145–0.583]). Patients with optimal levels of all three modifiable risk factors (SBP ≥ 140 mmHg, obesity with central obesity, inactivity) had a 71% lower risk of incident AF compared with participants with the least favorable risk factor profile (three MRFs). These results were similar to those using the baseline model, where participants with ≤ 1 MRF had a lower risk of incident AF compared with participants with three MRFs. In addition, compared to participant with maintained MRFs ≥ 2 to the next follow-up visit, participants with maintained or achieved MRFs ≤ 1 had significantly decreased risk of AF (hazard ratio [95% CI] for the number of MRFs ≤ 1 to ≤ 1, 0.328 [0.159–0.674]; and for the number of MRFs ≥ 2 to ≤ 1, 0.493 [0.341–0.713]) (Table 4). Likewise, compared to participant with an increased number of MRFs from ≤ 1 to ≥ 2, participants with maintained or achieved MRFs ≤ 1 had significantly decreased risk of AF (hazard ratio [95% CI] for the number of MRFs ≤ 1 to ≤ 0.1, 0.424 [0.199–0.906]; and for the number of MRFs ≥ 2 to ≤ 1, 0.638 [0.413–0.986]).

**Table 4:** Reduction of AF risk according to the change in number of modifiable risk factor.

Change in number of MRFs at every visits	No. of observation periods	No. of events	Multivariable-Adjusted HR (95% CI)*	*P*-value
Reference ≥ 2 → ≥ 2	7414	42		
≤ 1 → ≥ 2	4766	26	0.772 (0.472–1.262)	0.302
≥ 2 → ≤ 1	4781	14	0.493 (0.341–0.713)	0.002
≤ 1 → ≤ 1	36,447	100	0.328 (0.159–0.674)	< 0.001
Reference ≤ 1 → ≥ 2				
≥ 2 → ≥ 2			1.295 (0.792–2.117)	0.302
≥ 2 → ≤ 1			0.638 (0.413–0.986)	0.043
≤ 1 → ≤ 1			0.424 (0.199–0.906)	0.027

HR, hazard ratio; CI, confidence interval.

*Multivariable adjustment was for sex, area and the time-varying assessment of age, systolic blood pressure, chronic kidney disease, cardiovascular disease, HbA1c and total cholesterol.

### Impact of modifiable risk factor burden on the risk of AF

**Figure 2:**
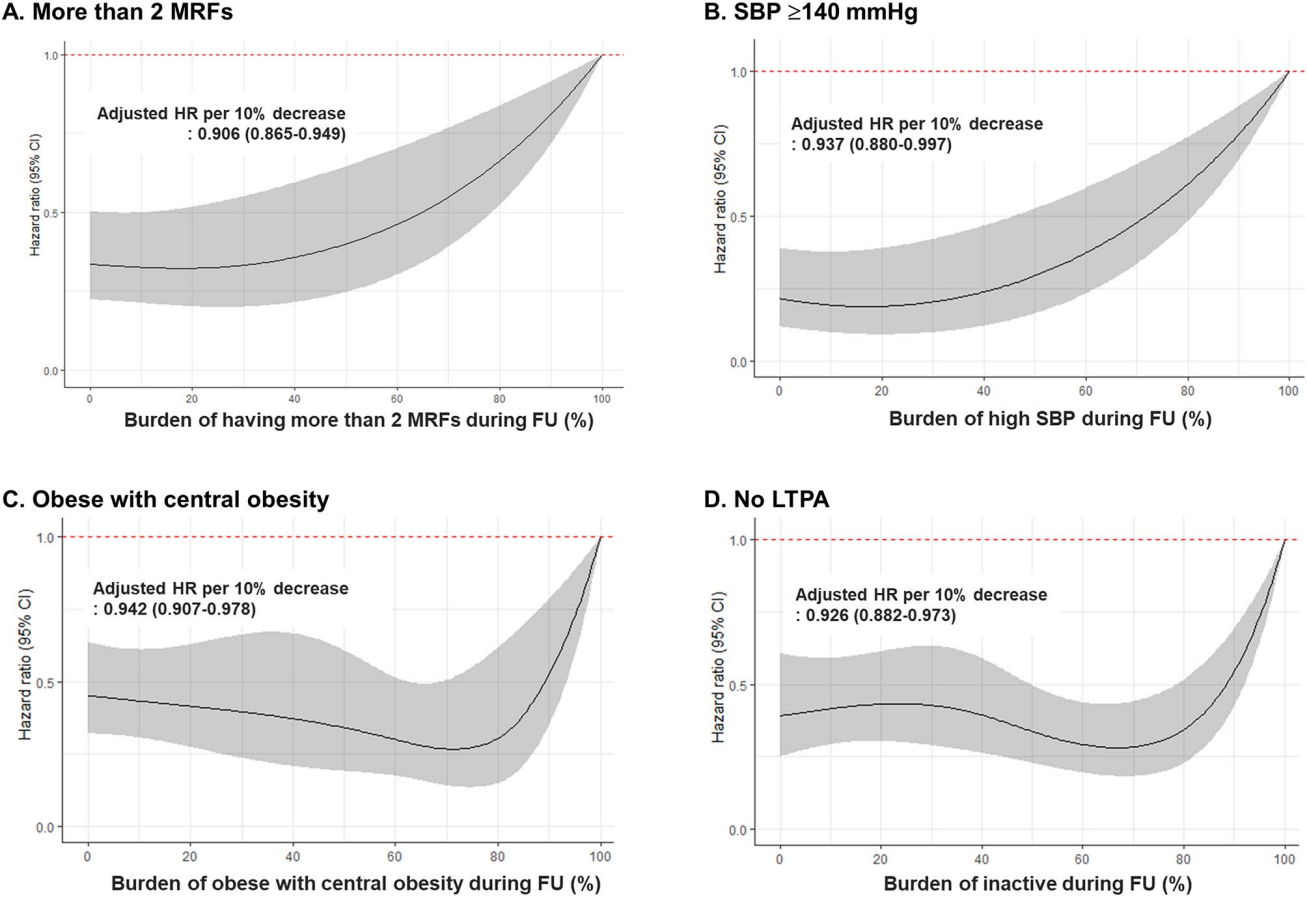
Associations of burden of having more than 2 MRFs (**A**), high SBP (**B**), obese with central obesity (**C**) and inactive (**D**) with risk of AF during follow-up. The solid black line and shaded gray areas represent hazard ratio and 95% confidence bands. Restricted cubic splines with 5 knots located at the 5th, 25th, 50th, 75th and 95th percentiles for hazard ratios were calculated with a burden of 100% as a reference. MRFs = modifiable risk factors; SBP = systolic blood pressure; LTPA = leisure time physical activity; HR = hazard ratio; CI = confidence interval; AF = atrial fibrillation.

Figure 2 depicts the risk of AF associated with continuous measures of MRFs burden during follow-up, using restricted cubic spline analysis (with a burden of 100% as reference). The proportion of visits with more than two MRFs (burden of more than two MRFs) was significantly associated with the risk of AF (hazard ratio [95% CI] per 10% decrease, 0.906 [0.865–0.949] (Fig. 2A). in particular, when the burden of more than two MRFs is less than two-thirds, the risk of AF decreased more than 50%. Similarly, a decrease in the proportion of each MRFs exposure continuously decreased the adjusted risk of AF (hazard ratio [95% CI] per 10% decrease for SBP ≥ 140 mmHg, 0.937 [0.880–0.997]; for obesity with central obesity, 0.942 [0.907–0.978]; for inactivity, 0.926 [0.882–0.973]). In addition, MRFs burden < 72% for high SBP, 89% for obesity with central obesity, and 88% for inactivity lowered the risk of incident AF by more than 50%.

## Discussion

This study had three principal findings. Firstly, in a time-updated model, three MRFs (SBP ≥ 140 mmHg, obesity with central obesity, and inactivity) were significant in the midlife general population. Secondly, participants who maintained or achieved an optimal risk factor profile had a significantly reduced risk of AF. These findings highlight the potential population and individual level impact of the number of MRFs on AF risk. Thirdly, decreased MRFs burden was associated with a decreased risk of AF. Our study shows that reducing the MRFs burden and maintenance or achievement of MRFs ≤ 1 plays a crucial role in reducing the risk of AF.

Previous studies have reported that the independent risk factors for incident AF include aging, hypertension, congestive heart failure, coronary artery disease, valvular heart disease, diabetes mellitus, male sex, obesity, and excessive alcohol use^8,12^. Furthermore, increased numbers of unhealthy lifestyle factors, including current smoking, heavy drinking (30 g/day) and lack of regular exercise, were associated with a higher risk of incident AF^18^. These risk factors play a crucial role in abnormal atrial remodeling, disease progression, and recurrence^19^. The Asian population tends to have higher percentages of abdominal fat with lower BMI compared with the European population^20^. Obesity and abdominal obesity were associated with AF risk^21–23^; however abdominal obesity is a risk factor for AF in nonobese Asian populations, but not in obese^21^. Thus, assessments of the combined impact of obesity and abdominal obesity on the risk of AF are important. Therefore, we used the combination of obesity and abdominal obesity to find the MRFs for AF risk. The current study showed that an SBP of more than 140 mmHg, obesity with central obesity, inactivity for leisure time, aging, male sex, and CVD were significant risk factors for AF incidence. Particularly, 81.2% of all participants had at least one MRFs, and 72.2% (n = 6535) had leisure-time inactivity. In addition, 28.7% of incident AF appears to be attributable to these three MRFs, with inactivity during leisure time being the greatest contributing risk factor, indicating the importance of MRFs management, especially increasing LTPA.

Although studies for risk prediction and treatment of AF have been extensive, AF prevention has received relatively little attention^7–10^. Current international guidelines recommend modifying an inappropriate diet, quitting smoking, abstaining from alcohol and recreational drugs, and participating in regular physical activity programs as key health behaviors to prevent the development of AF^10^. Our results showed that maintaining or achieving MRFs ≤ 1 was significantly associated with decreased risk of AF. In particular, we identified a consistent decrement in AF risk with progressively optimal risk factor profiles, with a striking 71% lowering of AF risk with optimal levels of MRFs (reversing SBP of more than 140 mmHg, obesity with central obesity and inactivity for leisure time). Our study suggests that risk factor improvement may decrease AF risk in general population. Similarly, Du X et al. reported that a high proportion of AF can be prevented by combining strategies, focusing on the high-risk population for better risk factor management, and emphasizing healthy lifestyle choices in the whole population^6^. Our findings indicate that it is possible to prevent approximately 29% of AF cases through risk factor modification. Unfortunately, we did not find significant associations between MRFs combinations and AF risk, as the sample size was too small and statistical power too low to analyze these outcomes. However, we believe that our findings provide firmer evidence to establish strategies for AF prevention in the general population.

Although the Framingham Heart Study reported that the risk factor burden, comprising modifiable risk factors, and having multiple morbidities play a crucial role in the lifetime risk of AF, associations between MRFs burden and incidence of AF have not been previously reported^24^. Our findings showed that the risk of AF progressively decreases according to the decrease in the proportion of visits with more than two MRFs during the follow-up period. These results indicate that even if the durations of exposure to MRFs are the same, the risk of AF may be lower in those who have a longer period of non-exposure to MRFs. We suggest that lengthening the period of non-exposure to MRFs (especially when the number of MRFs is one or less) during a lifetime could help reduce the risk of AF. Moreover, a log-linear association of high SBP burden with AF incidence suggests that there are cumulative effects of high SBP on the risk of AF. Therefore, minimizing the MRFs burden by early intervention and control could reduce the incidence of AF. There is also potential for the burden and costs of AF to be reduced. Our findings provide a necessary evidence base to support future investment in intervention trials aimed at modification of risk factors for AF in the general population without AF.

### Strengths and limitations

This study had several limitations that need to be addressed. First, the study population comprised healthy and middle-aged participants recruited from two specific communities in Korea (Ansan and Ansung). Thus, the PAF estimated in this study, which is population-specific, may not be applicable to the general Korean population. Second, self-reporting questionnaires may not have accurately reflected the level of LTPA. In addition, the LTPA was divided into only two groups (inactive vs. active), because the majority of the study population had 0 min/week of LTPA (inactivity) and a small event size when categorizing the active group. Thus, we could not assess the association between LTPA intensity and incidence of AF. Third, although smoking and drinking were not included due to missing data, similar results were found even after adjustment by adding smoking and drinking variables (Table S3). Fourth, it is possible that some paroxysmal and intermittent AF cases could have been missed since we did not use long-term electrocardiography (ECG) recording. Instead, we additionally analyzed the incidence of AF using the Korean Classification of Diseases-7 (KCD-7) codes, which are similar to the International Classification of Diseases-10 codes, for 7,620 participants who consented to data linkage between KoGES and the Korean National Health Insurance Service database. We confirmed that the overall incidence rate of AF using KCD-7 codes was 2.5%, which was similar to our results (2.0%). However, our current data still underestimate the true incidence of AF.

However, the study had several strengths. The main strengths were its community-based prospective design and the long follow-up period. In addition, to our knowledge, this is the first study to investigate the association between MRFs for incident AF using a time-updated model and assessing the cumulative effects of MRFs burden on AF risk in South Korea.

## Conclusions

In a prospective cohort study in Korea, our findings provide support for the concept that targeting MRFs, including high SBP, obesity with central obesity, and inactivity for leisure-time, has the potential to significantly reduce the individual risk and population burden of AF. Future studies on appropriate level of control of MRFs may provide insights into an efficient approach to reduce AF risk or burden in the general population. Presently, this needs to be scrutinized by prospective intervention trials to find suitable levels of control of MRFs.

## Methods

### Data source and study population

Data were obtained from the Ansan-Ansung cohort within the Korean Genome and Epidemiology Study (KoGES) which is conducted by the Korea National Institute of Health. The Ansan-Ansung cohort is an ongoing, prospective, community-based cohort study that was initiated in 2001–2002. The aim of this cohort study is to ascertain the relationships between genetic, environmental, and lifestyle determinants of chronic diseases such as diabetes mellitus, cerebrovascular disease, and hypertension in Korean people^25^. The participants are residents of both urban (Ansan) and rural (Ansung) areas. Enrolment in the study was based on the characteristics of the communities and the most efficient method of recruitment of a representative sample of the Korean population. Initially, the cohort comprised 10,030 participants that were 40–69 years old between 2001 and 2002. Follow-up examinations were conducted biennially between 2003 and 2018. All the participants provided written informed consent. The study protocol was approved by the Institutional Review Board of the Korea Centers for Disease Control and Prevention. All research procedures were performed in accordance with the relevant guidelines and regulations.

In the current study, participants with AF at baseline (n = 39), those with missing values for ECG at baseline (n = 12), those without ECG tests during follow-up (n = 55) and those who had no follow-up after baseline (n = 875) were excluded from the analysis (Fig. S1). The final study cohort included 9049 participants.

### Case ascertainment and follow-up

All participants underwent the 12-lead standard ECG at every visit to identify AF. The mortality data for participants lost to follow-up was ascertained by examination of National Death Records. The participants in this study cohort were followed until the index date (AF, death, or end of the study period [December 31, 2018], whichever came first). The primary outcomes were incident AF or AF-related mortality.

### Risk factor ascertainment

Information on the presence of CVD, including myocardial infarction, coronary artery disease, congestive heart failure and stroke/transient ischemic attack, were obtained using a questionnaire at every visit. The questionnaires were administered by trained interviewers according to a specified protocol. Blood pressure was measured using a mercury sphygmomanometer, by trained examiners, at least twice at the level of the heart, in a sitting position, and averaged. SBP was classified into three categories: < 120 mmHg, 120–139 mmHg, and ≥ 140 mmHg. Body mass index (BMI) was calculated as body mass in kilograms divided by height in meters squared. WC was measured at the mid-point between the lower ribs and the top of the iliac crest in the standing position. Central obesity was defined as a WC ≥ 85 cm in women and ≥ 90 cm in men^20^. Obesity was defined as a BMI ≥ 25 kg/m^2^, in accordance with the World Health Organization criteria for individuals of Asian descent^26^. Estimated glomerular filtration rate (eGFR) was calculated using the Chronic Kidney Disease Epidemiology Collaboration Study equation, and chronic kidney disease (CKD) was defined as an eGFR ≤ 60 mL/min/1.73 m^2^. LTPA including aerobics, jogging, swimming, tennis, golf, bowling, fitness club exercise, walking, and climbing was assessed using a questionnaire to quantify activities in the leisure time domain. All participants were asked about the types, duration, and frequency of their LTPA. LTPA was then categorized into no physical activity (inactivity) and > 0 min/weeks (active).

### Statistical analysis

Baseline characteristics, according to incident AF at the index date, were captured. Continuous variables were expressed as mean ± standard deviation and compared using t-tests; categorical variables were expressed as frequency (percentage) and compared using chi-square tests. Incidence rates of AF were reported as number of patients per 100,000 person-years.

We constructed time-varying Cox models with time-varying assessment of risk factors to identify significant MRFs for incident AF. Each participant contributed with two or more observation periods and each period lasted from one measurement until the next (up to eight times of measurements were used). The 9,049 participants contributed with 53,408 observation periods in the time-varying cox regression analysis for risk of AF. Models were initially adjusted for sex, area, and time-varying assessment of age, SBP, combinations of obesity and central obesity, LTPA, CKD, CVD, HbA1c and total cholesterol. To estimate the joint risk reduction associated with the change in number of MRFs we constructed a risk factor score composed of significant MRFs (one point each for: SBP ≥ 140 mmHg, obesity with central obesity and inactivity). Using Cox models with and without time-varying assessment of risk factors (Fig. S2), we compared multivariable-adjusted hazard ratios (HR) for participants with the least favorable risk factor profile (three points) to those with progressively favorable profiles (two, one, and zero points). To find the impact of the change in number of MRFs on the risk of AF, we conducted Cox proportional hazard regression analysis using time-varying assessment of the changes in number of MRFs at every visits. Time-varying change in number of MRFs to the next follow-up visit was classified into four groups, as follows: (1) ≥ 2 →  ≥ 2, (2) ≤ 1 →  ≥ 2, (3) ≥ 2 →  ≤ 1, (4) ≤ 1 →  ≤ 1. The proportional hazards assumption was tested using the scaled Schoenfeld residuals^27^. For all the above-mentioned outcomes, we calculated the population attributable fractions (PAF), which reflect the fraction of the event rate or risk, in a given period, attributable to the exposure of interest (assuming a causal relationship). The PAF were computed using indirect standardization using the SAS procedure STDRATE.

To investigate the cumulative effect of MRFs on AF risk, we assessed the association with each participant’s MRFs burden during follow-up. In this study, MRFs burden was defined as the proportion of times MRFs appeared during follow-up, based on the number of visits (Fig. S3). An adjusted model using a restricted cubic spline with 5 knots, located at the 5th, 25th, 50th, 75th and 95th percentiles, was constructed to flexibly display the association between the hazards of developing the outcome and MRFs burden, using a burden of 100% as a reference.

All statistical tests were two-tailed and *p*-values < 0.05 were considered statistically significant. All statistical analyses were performed using SAS software (ver. 9.4; SAS Institute, Cary, NC, USA) and R 3.5.3 (R Foundation, Vienna, Austria).

## Supplementary Information

Supplementary Figure 1.

Supplementary Figure 2.

Supplementary Figure 3.

Supplementary Table 1.

Supplementary Table 2.

Supplementary Table 3.

## Data Availability

The KoGES data will be made available following the submission of an application form, together with documents such as a research plan and IRB approval form, to the Korea Centers for Disease Control and Prevention (KCDC). The relevant data request process and contact information can be found by following this link: http://www.nih.go.kr/contents.es?mid=a50401010400#menu4_1_2.
