# Supplementary figures and images for: Lower number of modifiable risk factors was associated with reduced atrial fibrillation incidence in an 18-year prospective cohort study
Source: Sci Rep. 2022 Jun 2;12:9207. doi: 10.1038/s41598-022-13434-4 (PMC9163060; doi:10.1038/s41598-022-13434-4)

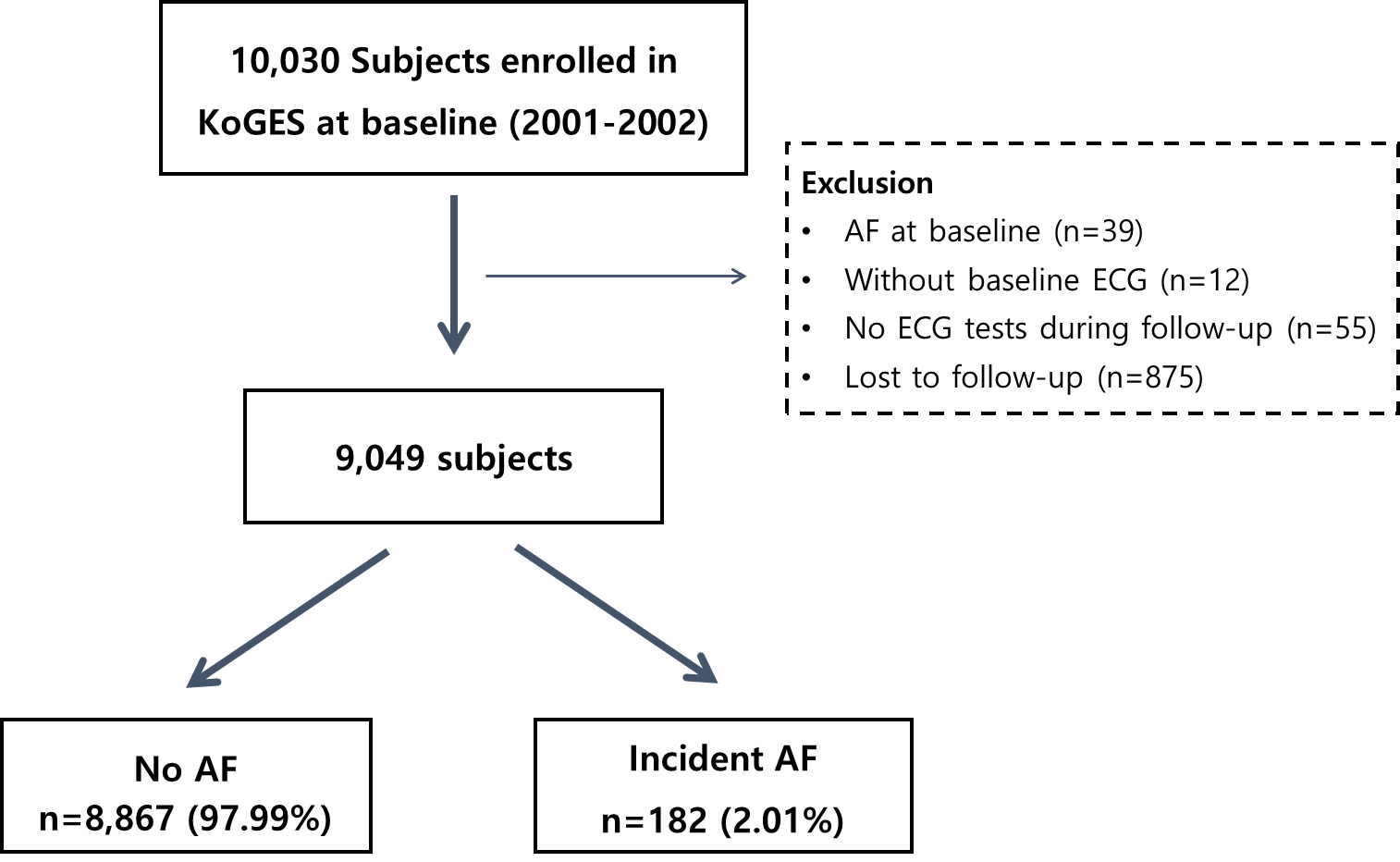


**Figure S1**. Study population

Supplement: Supplementary file 1 — Supplementary Figure 1. [file 41598_2022_13434_MOESM1_ESM.docx]
