# Supplementary material for: Lower number of modifiable risk factors was associated with reduced atrial fibrillation incidence in an 18-year prospective cohort study
Source: Sci Rep. 2022 Jun 2;12:9207. doi: 10.1038/s41598-022-13434-4 (PMC9163060; doi:10.1038/s41598-022-13434-4)
Supplement: Supplementary file 2 — Supplementary Figure 2. [file 41598_2022_13434_MOESM2_ESM.docx]

**
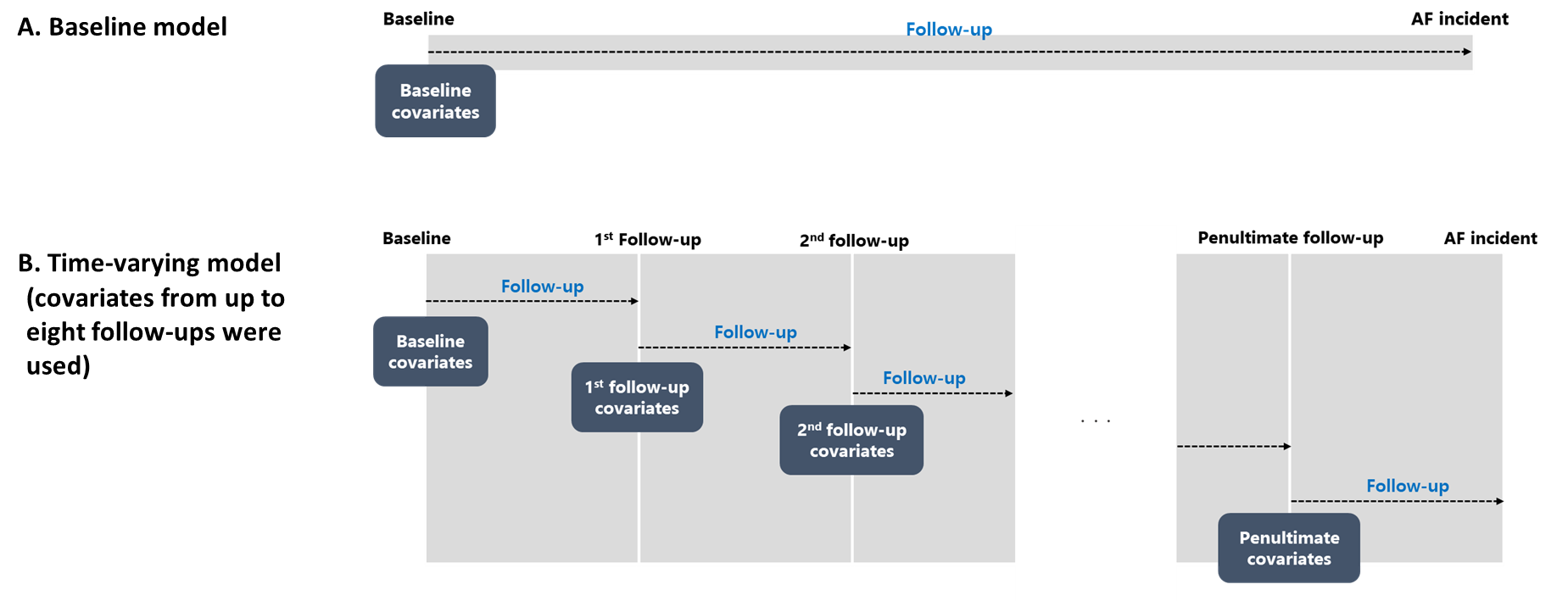
**

**Figure S2.** Baseline and time-updated Cox regression models incorporating the changes in MRF over time and the estimated MRF burden. A. Baseline model and B. Time-updated model
