# Supplementary material for: Lower number of modifiable risk factors was associated with reduced atrial fibrillation incidence in an 18-year prospective cohort study
Source: Sci Rep. 2022 Jun 2;12:9207. doi: 10.1038/s41598-022-13434-4 (PMC9163060; doi:10.1038/s41598-022-13434-4)
Supplement: Supplementary file 3 — Supplementary Figure 3. [file 41598_2022_13434_MOESM3_ESM.docx]

**
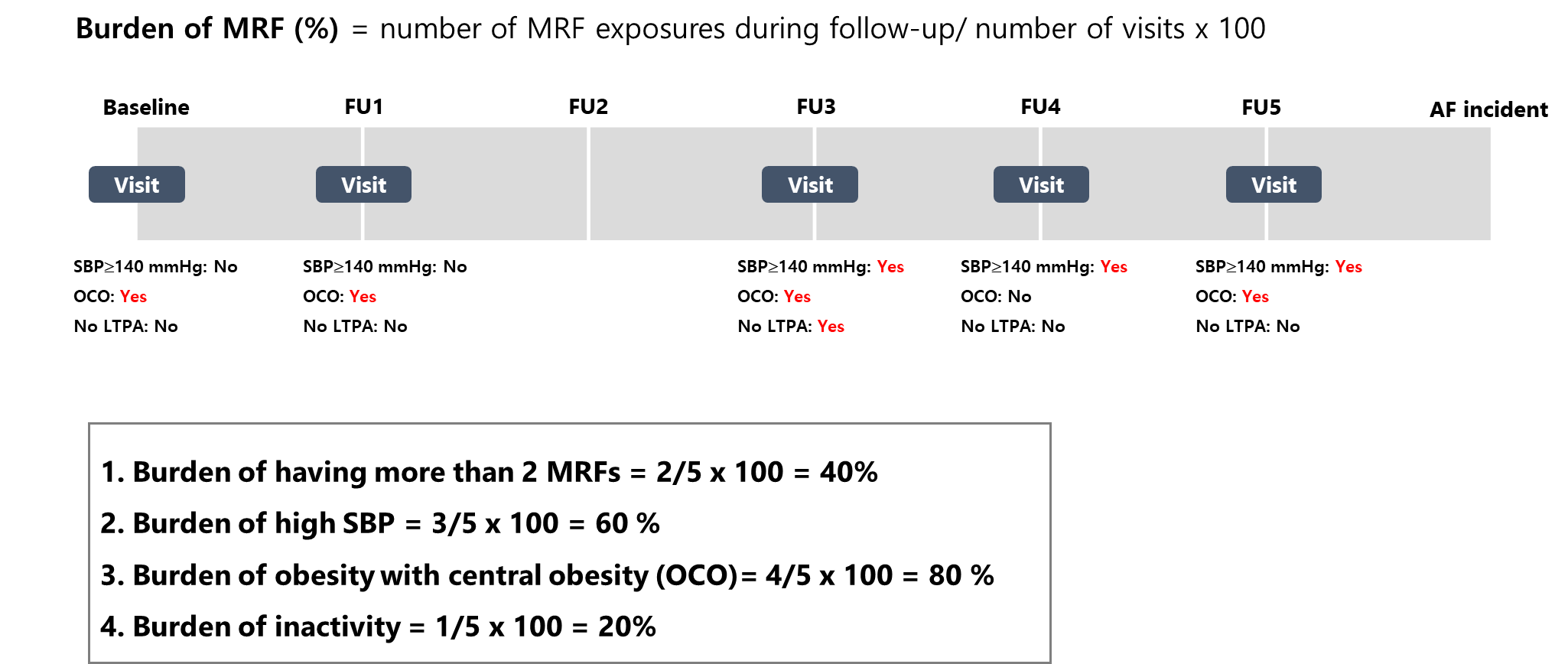
**

**Figure S3.** Estimation of MRF burden. Modifiable risk factors include time-varying systolic blood pressure ≥140 mmHg, obesity with central obesity, and inactivity. High SBP represents SBP ≥140 mmHg; AF indicates atrial fibrillation; SBP, systolic blood pressure; MRF, modifiable risk factors; OCO, obesity with central obesity; FU, follow-up.
