# Supplementary material for: Lower number of modifiable risk factors was associated with reduced atrial fibrillation incidence in an 18-year prospective cohort study
Source: Sci Rep. 2022 Jun 2;12:9207. doi: 10.1038/s41598-022-13434-4 (PMC9163060; doi:10.1038/s41598-022-13434-4)
Supplement: Supplementary file 4 — Supplementary Table 1. [file 41598_2022_13434_MOESM4_ESM.docx]

**Table S1**. Incidence rates of atrial fibrillation per 100,000 person-years during follow-up period by age and sex

|  | **2003-2004** | | |  | **2005-2006** | | |  | **2007-2008** | | |  | **2009-2010** | | |  | **2011-2012** | | |  | **2013-2014** | | |  | **2015-2016** | | |  | **2017-2018** | | |
| --- | --- | --- | --- | --- | --- | --- | --- | --- | --- | --- | --- | --- | --- | --- | --- | --- | --- | --- | --- | --- | --- | --- | --- | --- | --- | --- | --- | --- | --- | --- | --- |
|  | **Event** | **PY** | **IR** |  | **Event** | **PY** | **IR** |  | **Event** | **PY** | **IR** |  | **Event** | **PY** | **IR** |  | **Event** | **PY** | **IR** |  | **Event** | **PY** | **IR** |  | **Event** | **PY** | **IR** |  | **Event** | **PY** | **IR** |
| **Overall** | 17 | 15441.20 | 110.10 |  | 36 | 31233.14 | 115.26 |  | 55 | 45346.06 | 121.29 |  | 82 | 60789.16 | 134.89 |  | 109 | 73514.18 | 148.27 |  | 130 | 87299.98 | 148.91 |  | 151 | 104513.33 | 144.48 |  | 182 | 118531.04 | 153.55 |
| **Age group**, yrs |  |  |  |  |  |  |  |  |  |  |  |  |  |  |  |  |  |  |  |  |  |  |  |  |  |  |  |  |  |  |  |
| 40-49 | 3 | 7139.31 | 42.02 |  | 5 | 14639.40 | 34.15 |  | 9 | 21397.22 | 42.06 |  | 15 | 28912.17 | 51.88 |  | 21 | 35228.98 | 59.61 |  | 25 | 42425.15 | 58.93 |  | 30 | 50799.40 | 59.06 |  | 35 | 57626.24 | 60.74 |
| 50-59 | 4 | 4124.67 | 96.98 |  | 10 | 8368.46 | 119.50 |  | 18 | 12192.88 | 147.63 |  | 25 | 16352.85 | 152.88 |  | 36 | 19806.12 | 181.76 |  | 41 | 23469.81 | 174.69 |  | 47 | 28334.88 | 165.87 |  | 56 | 32133.99 | 174.27 |
| 60-69 | 10 | 4177.21 | 239.39 |  | 21 | 8225.27 | 255.31 |  | 28 | 11755.96 | 238.18 |  | 42 | 15524.15 | 270.55 |  | 52 | 18479.08 | 281.40 |  | 64 | 21405.03 | 299.00 |  | 74 | 25379.05 | 291.58 |  | 91 | 28770.81 | 316.29 |
| **Sex** |  |  |  |  |  |  |  |  |  |  |  |  |  |  |  |  |  |  |  |  |  |  |  |  |  |  |  |  |  |  |  |
| Male | 12 | 7337.63 | 163.54 |  | 25 | 14822.01 | 168.67 |  | 38 | 21509.68 | 176.66 |  | 56 | 28726.63 | 194.94 |  | 71 | 34628.83 | 205.03 |  | 83 | 41012.32 | 202.38 |  | 98 | 48878.87 | 200.50 |  | 122 | 55052.79 | 221.61 |
| Female | 5 | 8103.56 | 61.70 |  | 11 | 16411.13 | 67.03 |  | 17 | 23836.38 | 71.32 |  | 26 | 32062.53 | 81.09 |  | 38 | 38885.35 | 97.72 |  | 47 | 46287.66 | 101.54 |  | 53 | 55634.45 | 95.26 |  | 60 | 63478.24 | 94.52 |

PY, person-years; IR, incidence rate

Events were reported as cumulative incident in that year.
