# Supplementary material for: Lower number of modifiable risk factors was associated with reduced atrial fibrillation incidence in an 18-year prospective cohort study
Source: Sci Rep. 2022 Jun 2;12:9207. doi: 10.1038/s41598-022-13434-4 (PMC9163060; doi:10.1038/s41598-022-13434-4)
Supplement: Supplementary file 5 — Supplementary Table 2. [file 41598_2022_13434_MOESM5_ESM.docx]

**Table S2**. Incidence rate of AF per 100,000 person-years according to the number of MRF at baseline

| **Number of MRF**  **at baseline** | **N** | **Cases (%)** | **PY** | **IR per 100,000 PY (95% CI)** |
| --- | --- | --- | --- | --- |
| 3 | 408 | 16 (3.9) | 5203.6 | 307.5 (175.8-475.5) |
| 2 | 2033 | 51 (2.5) | 25875.1 | 197.1 (146.8-254.8) |
| 1 | 4909 | 90 (1.8) | 64620.8 | 139.3 (112.0-169.5) |
| 0 | 1699 | 25 (1.5) | 22831.6 | 109.5 (70.91-56.4) |

Modifiable risk factors include systolic blood pressure ≥140 mmHg, obesity with central obesity, and inactivity. MRF, modifiable risk factors; IR, incidence rate; PY, person-years.
