# Supplementary material for: Lower number of modifiable risk factors was associated with reduced atrial fibrillation incidence in an 18-year prospective cohort study
Source: Sci Rep. 2022 Jun 2;12:9207. doi: 10.1038/s41598-022-13434-4 (PMC9163060; doi:10.1038/s41598-022-13434-4)
Supplement: Supplementary file 6 — Supplementary Table 3. [file 41598_2022_13434_MOESM6_ESM.docx]

**Table S3**. Risk factors for incident atrial fibrillation using a time-updated model

|  | **Multivariable-Adjusted**  **HR (95% CI)*** | **P-value** |
| --- | --- | --- |
| **Number of MRF** |  |  |
| 3 | Reference |  |
| 2 | 0.739 (0.361-1.515) | 0.409 |
| 1 | 0.447 (0.221-0.903) | 0.025 |
| 0 | 0.286 (0.134-0.611) | 0.001 |
| **Age,** ≥70 years | 1.636 (1.069-2.503) | 0.023 |
| **Sex**, male | 2.260 (1.359-3.759) | 0.002 |
| **Smoking** |  |  |
| Never | Reference |  |
| Prior | 1.326 (0.817-2.152) | 0.254 |
| Current | 1.034 (0.609-1.756) | 0.902 |
| **Drinking** |  |  |
| Never | Reference |  |
| Prior | 1.005 (0.532-1.897) | 0.988 |
| Current | 1.119 (0.751-1.668) | 0.580 |
| **Cardiovascular disease** | 2.027 (1.313-3.129) | 0.001 |
| **Chronic kidney disease** | 1.086 (0.721-1.637) | 0.692 |
| **HbA1c** | 1.037 (0.643-1.670) | 0.882 |
| **Total cholesterol** | 0.732 (0.515-1.042) | 0.084 |

HR=hazard ratio; CI=confidence interval; LTPA=leisure time physical activity

*Multivariable adjustment was for sex, area and time-updated assessment of age, diatolic blood pressure, combinations of obesity and central obesity, LTPA, smoking, drinking, chronic kidney disease, cardiovascular disease, HbA1c and total cholesterol.
